# Coelenterazine-Dependent Luciferases as a Powerful Analytical Tool for Research and Biomedical Applications

**DOI:** 10.3390/ijms21207465

**Published:** 2020-10-10

**Authors:** Vasilisa V. Krasitskaya, Eugenia E. Bashmakova, Ludmila A. Frank

**Affiliations:** 1 Institute of Biophysics SB RAS, Federal Research Center “Krasnoyarsk Science Center SB RAS”, 660036 Krasnoyarsk, Russia; vasilisa.krasitskaya@gmail.com (V.V.K.); jeyn_a@bk.ru (E.E.B.); 2 School of Fundamental Biology and Biotechnology, Siberian Federal University, 660041 Krasnoyarsk, Russia

**Keywords:** bioluminescence, coelenterazine, luciferase, Ca^2+^-regulated photoprotein, analytical systems

## Abstract

The functioning of bioluminescent systems in most of the known marine organisms is based on the oxidation reaction of the same substrate—coelenterazine (CTZ), catalyzed by luciferase. Despite the diversity in structures and the functioning mechanisms, these enzymes can be united into a common group called CTZ-dependent luciferases. Among these, there are two sharply different types of the system organization—Ca^2+^-regulated photoproteins and luciferases themselves that function in accordance with the classical enzyme–substrate kinetics. Along with deep and comprehensive fundamental research on these systems, approaches and methods of their practical use as highly sensitive reporters in analytics have been developed. The research aiming at the creation of artificial luciferases and synthetic CTZ analogues with new unique properties has led to the development of new experimental analytical methods based on them. The commercial availability of many ready-to-use assay systems based on CTZ-dependent luciferases is also important when choosing them by first-time-users. The development of analytical methods based on these bioluminescent systems is currently booming. The bioluminescent systems under consideration were successfully applied in various biological research areas, which confirms them to be a powerful analytical tool. In this review, we consider the main directions, results, and achievements in research involving these luciferases.

## 1. Introduction

**Figure 1:**
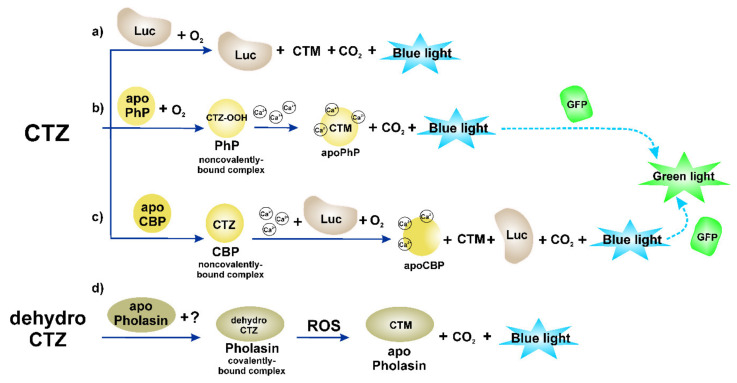
Variety of coelenterazine-dependent bioluminescent systems. (**a**–**d**) pathways of coelenterazine oxidation with the release of blue light, found in diverse organisms. Luc—luciferase; apoPhP—apophotoprotein; apoCBP—apo-coelenterazine binding protein; CTZ and CTM—coelenterazine and coelenteramide. Explanations are given in the text.

It would not be an exaggeration to say that most of the currently known luminous organisms are marine inhabitants. These are representatives of different taxa, with bioluminescent systems of various degrees of complexity, with different mechanisms of bioluminescent reactions. Noteworthy here is the fact that in most of these bioluminescent systems, the compounds with similar chemical structures containing imidazopyrazinone skeleton act as a substrate (luciferin). As such, coelenterazine (CTZ) is most deeply and frequently studied (Figure 1). The history of its discovery, information on occurrence, properties and reactions are presented in the book by O. Shimomura—recently reedited [1]. Bioluminescence reaction arises when luciferin is oxidized by the molecular oxygen catalyzed by a specific enzyme, luciferase (Luc). The CTZ-dependent luciferases are represented by two strictly different types—luciferases and photoproteins—whose bioluminescence is dependent on Ca^2+^ (the so-called Ca^2+^-regulated photoproteins, PhP). The first ones catalyze CTZ oxidation in accord with the classical enzyme–substrate interaction yielding coelenteramide (CTM, the decarboxylated derivative of CTZ), CO_2_, and a quantum of blue light (Figure 1a). Luciferases of many organisms are well studied, their cDNAs were cloned, and the recombinant analogues as well as many genetically modified variants with the new improved properties were obtained [2,3].

The CTZ oxidation by polypeptide apophotoprotein (apoPhP) results in the formation of a stable complex comprising a protein and 2-hydroperoxycoelenterazine, which is actually a Ca^2+^-regulated photoprotein: bioluminescence occurs when calcium ions are bound (Figure 1b). The reaction products are: the complex of apoPhP with CTM and three Ca^2+^, CO_2,_ and a quantum of blue light. So, in opposition to luciferases capable of making many turnovers, like the other enzymes, photoproteins produce the flash-type light only once. This suggests a fundamentally linear relationship between the protein amount and the light emitted. Being the in situ stable enzyme–substrate complexes, photoproteins have become of great academic interest—many proteins of the wild type (WT) and their mutant variants with altered bioluminescent properties were obtained, tertiary structures were determined, and the reaction mechanism was studied (e.g., [4]).

Of interest is the CTZ-dependent bioluminescent system found in a soft coral *Renilla*. Its particular feature is that coelenterazine in this animal is safety packed into the Ca^2+^-dependent coelenterazine-binding protein (CBP) cavity (Figure 1c) [5]. Its oxidation catalyzed by Renilla luciferase takes place when calcium ions are bound to the CBP [6]. In the in vitro experiments, the CBP was found to be of higher bioluminescence efficiency as a substrate compared to the free CTZ, possibly due to the formation of the CBP-luciferase complex [7].

As might be presumed, such a variety of bioluminescent systems in terms of organization characteristics is associated with the need to localize the substrate in such a way as to ensure its presence at the right time and in the right place. It is a general opinion that bioluminescence is a vital adaptation selected for in evolution. As the membrane permeability of CTZ is rather high [8], this substrate can be distributed throughout the whole organism. To keep the bioluminescence system in a “ready for action” mode, in case (a) luciferase and CTZ are stored separately in special secretory glands and are injected into the water, where they mix, forming a luminous cloud to deceive or scare away a predator (for example, systems of copepods *Metridia* or *Gaussia*). In case (b), luciferase and preoxydized luciferin are stable molecules of photoprotein ready to act under Ca^2+^ control, whose concentration rises by nerve stimulation (e.g., systems of coelenterates *Aeqouria*, *Obelia*, *Beroe*, and others). In case (c), CTZ luciferin is stored inside the CBP complex and becomes available for oxidation also under the Ca^2+^ control (e.g., the system of soft coral *Renilla*). It should be noted that owing to the stability of coelenterazine localized in CBP to spontaneous oxidation, this protein has been proved to be useful at analytical application of this form of substrate [9,10,11].

**Figure 2:**
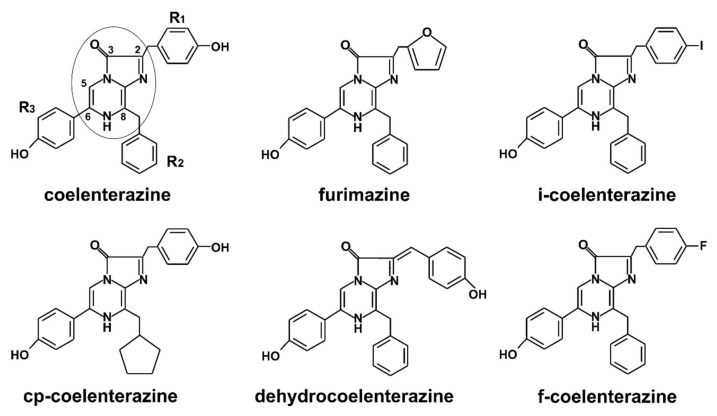
Chemical structures of coelenterazine and its analogues, mentioned in the review. Imidazopyrazinone core is shown in a circle.

There is another kind of photoprotein utilizing CTZ derivative as a substrate with a bioluminescent system significantly different from that of Ca^2+^-regulated photoproteins (Figure 1d). It was found in glowing squids *Watasenia scintillans* and *Symplectoteuthis oualaniensis* (symplectin) and bivalve mollusk *Pholas dactylus* (pholasin). To date, the structure and functioning of the system are still poorly understood [12]. It is now classified as a reactive oxygen species (ROS)-sensitive photoproteins comprising a glycoapoprotein (shown for apo-pholasin only) with covalently bound (through sulfhydryl group of one of the cysteine residues) dehydrocoelenterazine (see Figure 2) for the light-emitting source. Nevertheless, poor knowledge about this bioluminescent system organization did not prevent the development of pholasin application as an indicator of ROS in various fields of research.

All natural CTZ-dependent luciferases emit blue light with maxima at 460–480 nm, apparently from the fact that the emitter is the same coelenteramide (or its close derivatives) in an excited state [1]. However, bioluminescence systems of many coelenterates contain the re-emitted molecule, whose fluorescence is shifted to the longer wavelength part of the spectrum with a maximum at 510 nm. The green fluorescent protein (GFP) was discovered in 1961 in the jellyfish *Aequorea aequorea* [13] and then in other organisms (e.g., *Clytia* and *Renilla*). It was shown that the protein contains a chromophore inside the cylinder-shaped molecule, which is spontaneously formed from three amino acids (Ser65, Tyr66, and Gly67) [14,15]. So, light energy produced by CTZ-dependent luciferases is transferred to GFP resulting in the emission of green light. This process involves the mechanism of fluorescence (bioluminescence) resonance energy transfer (FRET/BRET) which underlies many modern research methods. After the cDNA of *Aequorea* GFP was cloned, and especially when it was successfully expressed in living organisms, its usefulness as an in vivo marker became apparent. The obvious importance of GFP for biomedical research activated the development of its recombinant forms with the improved properties (brighter, in different colors, etc.). Currently, the number of publications about this protein and its application is enormous. For a short account on the GFP chemistry, one can refer to the book by Shimomura [1].

The chemical structure of coelenterazine of natural type was determined, and its molecule was chemically synthesized in the mid-1960s–1970s [16]. The fundamental interest in the phenomenon of bioluminescence, its mechanism and the desire to change its characteristics resulted in the development of new synthetic variants of coelenterazine-like molecules. Some of these studies were aimed at searching those showing the improved bioluminescent signal. As of now, there are hundreds of CTZ variants which were synthesized and tested for applicability as the luciferase substrates. Of special interest were the CTZ variants providing alternative bioluminescence spectra and kinetics, being at the same time more stable under storage [17,18,19,20]. All coelenterazine analogues retain the molecule skeleton and differ in the structures of radicals R1-R3 (Figure 2). Therefore, the quantity of potentially useful synthetic CTZ variants is to be rather big, especially if it concerns the other luciferases. Many of CTZ variants demonstrate good perspectives for practical applications and are commercially available. The structures of some of them discussed hereafter are presented in Figure 2.

All CTZ-dependent luciferases known today, with a rare exception, are small, single-chain proteins, providing bright bioluminescence; their recombinant variants are easily available, and many of them are commercial products. These factors account for significant interest in their use as highly sensitive reporters in analytical systems for both in vitro and in vivo applications. Currently, there is a whole arsenal of luciferases as well as CTZ derivatives. These bioluminescent systems were successfully applied in various biological research areas, which confirms them to be a powerful analytical tool. It is impossible to cover a huge number of publications on this topic. In this review, we consider the main directions, results, and achievements in research involving these luciferases over the past 10–15 years.

## 2. Analytical Application of Ca^2+^-Regulated Photoproteins

The Ca^2+^-regulated photoproteins are single-chain globular proteins (MW of about 21 kDa) with a tightly but non-covalently bound molecule of pre-oxidized coelenterazine derivative—2-hydroperoxycoelenterazine in the protein internal cavity [21,22]. By virtue of this, their light emission is independent of the presence of molecular oxygen in the reaction mixture, this being one more feature of the Ca^2+^-regulated photoproteins that distinguishes them from other bioluminescent enzymes. Of all the known photoproteins, only aequorin from *Aeqourea victoria* and obelin from *Obelia longissima*, the most deeply studied ones, are used as reporters in analytics.

Bioluminescence reaction of this type of photoproteins is initiated by the binding of calcium ions to the EF-hand Ca^2+^-binding loops on the surface of a protein molecule, causing small conformational changes within the internal cavity of the protein. This disturbs the network of hydrogen bonds of the active center of the molecule and triggers the reaction of 2-hydroperoxycoelenterazine decarboxylation, yielding a mole of CO_2_ and the protein-bound product, coelenteramide, in an excited state. Its relaxation to the ground state is accompanied by light emission with λmax at 465–495 nm [23].

Cloning of cDNAs encoding several photoproteins and their expression in bacterial cells, as well as effective activation of the recombinant apophotoproteins with a synthetic coelenterazine (under calcium-free conditions in the presence of O_2_) opened the way to obtaining almost unlimited amounts of the recombinant proteins as well as to producing different mutated and chimeric photoproteins. Many photoprotein mutants with altered characteristics such as higher thermostability and bioluminescence activity, different emission color, faster or slower bioluminescence kinetics, and modified calcium affinity [3,4,24,25], as well as chimeric photoproteins genetically fused with different polypeptides, were produced. The diversity of photoprotein variants and synthetic coelenterazine analogues allow the development of novel bioluminescent assays or to improve characteristics of the already existing ones.

### 2.1. Ca^2+^-Regulated Photoproteins as Indicators of Intracellular Ca^2+^

**Figure 3:**
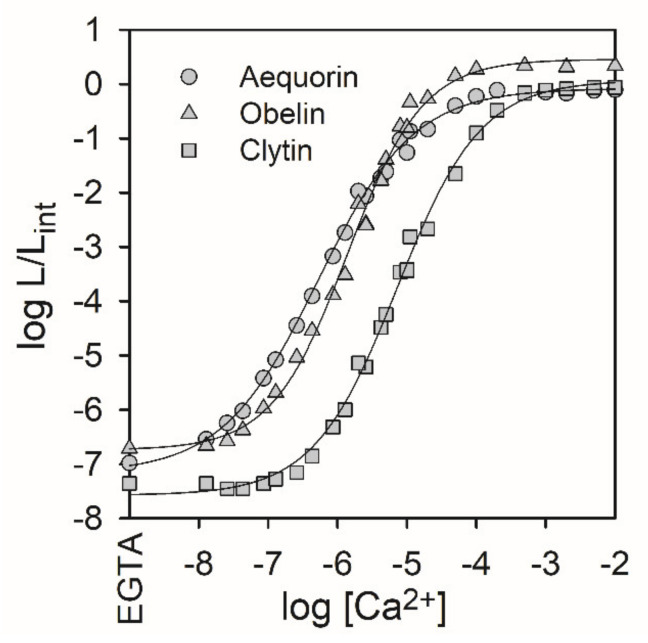
Ca2+ concentration—effect curves for recombinant photoproteins. L—light intensity at the particular Ca2+ concentration; Lint—total light intensity at saturating Ca2+ concentration.

The traditional application of Ca^2+^-regulated photoproteins is conditioned by their high specificity and sensitivity to calcium ions in a physiological concentration range (Figure 3) where they are used as a valuable probe for studying Ca^2+^ signaling [26]. Intracellular calcium ion signaling regulates numerous basic cellular processes including muscle contraction, release of mediators, synaptic plasticity, neuronal signal transmission, gene expression, proliferation, apoptosis, etc. Generally, calcium is often regarded as an agent affecting intracellular processes by changing its concentration—i.e., the level of free calcium, varying from 50 nM to about 10 μM at the peaks of a number of physiological signals [27]. Moreover, intracellular calcium signals regulate processes that operate over a wide time range, from neurotransmitter release at the microsecond scale to gene transcription, which lasts for minutes and hours. Thus, the time course, the amplitude, and, most notably, the local action site in well-defined cellular subcompartments are essential features of intracellular calcium signaling pathways.

Aequorin has been widely used as a gene-encoded Ca^2+^ indicator (GECI) in cells since it was cloned in 1985 and has been shown to be an effective tool to monitor intracellular [Ca^2+^] (concentration of Ca^2 +^) [28]. The emission of photoproteins is characterized by a high signal-to-noise ratio and a wide dynamic range. Bioluminescent recordings of calcium signals do not require external illumination, thus avoiding problems such as phototoxicity, photobleaching, autofluorescence, and undesirable stimulation of photobiological processes. Photoproteins have very low buffering capacity and are not toxic. Moreover, they can be modified to introduce specific targeting sequences providing selective routing of the photoprotein to a cell region of interest where local calcium transients need to be measured. Native-type aequorin is exclusively cytosolic, but the recombinant aequorin chimeras were constructed for targeting different intracellular compartments (nuclei, mitochondria, subplasma-membrane cytosol, endoplasmic/sarcoplasmic reticulum, Golgi apparatus, secretory vesicles, peroxisomes) [29,30].

The major efforts of researchers over the past 15 years have focused on expanding the arsenal of different photoprotein probes to cover specific requirements for visualization and quantitative estimation of the intracellular calcium signals. The main points here concerned: probe stability during in vivo measurements; the possibility to measure both low and high [Ca^2+^] in single cells, in cytoplasm, and within defined intracellular compartments; to also measure both fast and slow [Ca^2+^] fluxes; as well as the design of differently colored probes for multiplexing and red-colored probes for in vivo imaging.

Although recombinant photoproteins from various organisms are closely related, they differ in properties as intracellular calcium indicators with regard to Ca^2+^ concentration detection limit, Mg^2+^ sensitivity, and the rate of luminescence rise [31,32]. As a Ca^2+^-indicator, aequorin is more suitable at [Ca^2+^] ranging from 10^−7.5^ to 10^−4.5^ M, while obelin—at [Ca^2+^] between 10^−6.5^ and 10^−3.5^ M [31]. Clytin is the least sensitive to [Ca^2+^] (K_D_ = 500 nM) [31]; berovin responds to changes in calcium concentration starting below 10^−8^ M and reaches saturation at approximately 10^−4^ M [33]. Sensitivity to calcium of mnemiopsin isoform 2 is lower compared to berovin and hydromedusan photoproteins and extends from 10^−6^ to 10^–4.5^ M [34]. However, photoproteins of a wild type cannot be used as a probe for cellular organelles where [Ca^2+^] is much higher—e.g., in the lumen of endoplasmic or sarcoplasmic reticulum (about 300–500 μM) or in mitochondria that can reach values of up to 100 μM. Numerous studies on photoprotein EF-hand motif have shown that calcium affinity of the protein can be changed. Different approaches have been used to perform modifications: specific point mutations of the Ca^2+^-binding sites, random mutagenesis and functional screening, replacement of the consensus sequence of the first Ca^2+^-binding site with a loop sequence belonging to other EF-hand Ca^2+^-binding proteins [4,25], or/and the usage of coelenterazine synthetic analogues [31]. The aequorin variant with a reduced Ca^2+^ affinity was produced and proved to be eminently suitable for measuring Ca^2+^ within cell compartments with high calcium concentration (endoplasmic reticulum, mitochondria, etc.) [35,36].

Several studies have been devoted to modifying the emission spectrum of photoproteins for making them applicable for in vivo imaging in deep tissue and for multiplexing screening. It is well known that animal tissue absorbs and scatters less light in the 600–900 nm range. Many aequorin and obelin variants with a broadened emission spectrum and various decay kinetics have been obtained by site-directed mutagenesis of the coelenterazine-binding cavity or by random mutagenesis [4,25]. The incorporation of non-natural amino acids into cystein-free aequorin in combination with various coelenterazine analogues allowed the production of aequorin emitting in the yellow region of the spectrum (maximum at 526 nm) with long decay kinetics (half-life up to one minute) [37]. Another approach to obtain red-emitting photoproteins is to fuse them with fluorescent proteins or chemically attach them to fluorophores which act as BRET acceptors. For instance, aequorin has been conjugated to quantum-dot particles (CdSe/ZnS), resulting in the limited energy transfer with the presence of an emission peak around 540 nm [38]. The largest emission shift has been produced by fusing photoproteins with different variants of GFP (yellow, red, orange, etc.) (see, for review, [39]). The fusion of aequorin with a tandem dimer tdTomato generated “redquorin” characterized by high BRET efficiency, a major emission peak at 582 nm, and a sensitivity high enough to detect spontaneous Ca^2+^ oscillations in single cells [40]. Aequorins, fused with GFP and RFP (red fluorescent protein [41]) and targeted to different subcellular locations have been successfully used to simultaneously and independently record the changes in Ca^2+^ concentrations in different subcellular compartments of the same cells—cytosol, mitochondria, endoplasmic reticulum (ER), and nucleus [42,43]. Of note is that in these studies, the mutant aequorin with a low affinity for Ca^2+^, as well as an artificial variant of coelenterazine, f-CTZ (Figure 2), were used for [Ca^2+^] measurements in ER.

In the last 10 years, aequorin has been widely used as a Ca^2+^ indicator in a great variety of research: for studying the neural basis for *Drosophila* behavior [44,45,46,47,48,49]; exploring brain activity and visualizing Ca^2+^ signaling events that occur at the early stages of zebrafish development [44,50,51,52,53,54]; elucidating the Ca^2+^ response to biotic or abiotic stress in different plants [55,56,57,58,59,60,61,62] and yeast [63]; as well as for protozoa studies [64].

The extension of the arsenal of photoprotein probes has allowed to overcome some limitations concerning the application of photoproteins as probes for imaging Ca^2+^ fluxes in cell populations and in intact organisms as well as the measuring of [Ca^2+^] in single cells, in the cytoplasm, and other cell compartments.

### 2.2. Ca^2+^-Regulated Photoproteins as Effective Reporters for In Vitro Binding Assay

**Figure 4:**
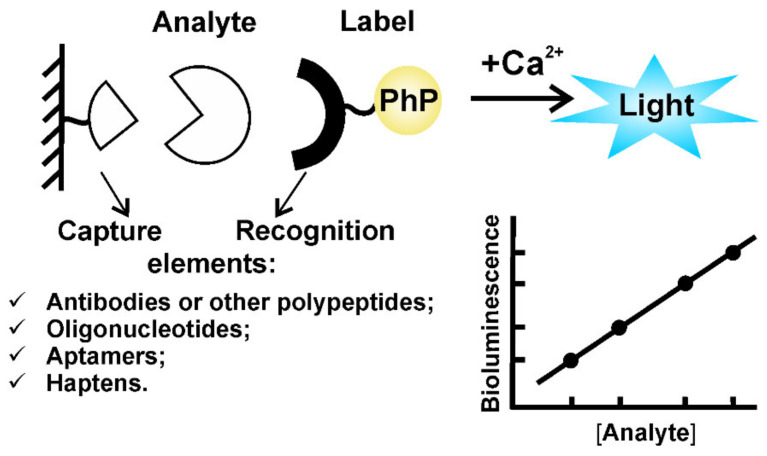
General scheme for bioluminescent solid-phase binding assay. Label is a chemically synthesized conjugate or genetically fused recognition element with a reporter molecule (PhP).

At saturating concentration of Ca^2+^, the photoprotein-induced signal reaches its maximum, and overlaps several logs of concentration with a linear relationship between photoprotein amount and bioluminescence. A high quantum yield of the reaction, a virtual absence of the background bioluminescent signal, and a high sensitivity of modern photometers make the photoprotein detection down to attomole level possible [65]. Aequorin and obelin have been tested as reporters in various binding assays (Figure 4), such as ELISA-type, homogeneous, DNA-hybridization assay, or whole-cell sensing systems and have always shown a very low detection limit. Photoproteins have been shown to be inherently endowed with distinct advantages over absorbance, fluorescent, or radioactive reporters for use as labels in bioassays in vitro [66,67].

The detection of multiple biomarkers is a model trend that has proved to be effective in precise medicine for diagnosis and management of the disease. Mutated variants of aequorin and obelin as well as “semi-synthetic” aequorins (aequorins with introduced synthetic coelenterazine analogues) displaying altered bioluminescent properties (emission maxima and kinetics) expanded the analytical applications of photoproteins and made them amenable to simultaneous multianalyte detection in a single well.

With site-directed mutagenesis of the amino acids in the obelin active site applied, two stable and active obelin mutants of different color and bioluminescence reaction rates, suitable for multiplex analysis, were produced. The signals of the mutants obtained were shown to be effectively separated by using wideband optic filters and temporal resolution [68,69].

Obviously, this approach is especially suitable when the diagnosis is determined by the balance between a pair of hormones—e.g., gonadotrope hormones, luteinizing (hLH), and follicle-stimulating (hFSH) ones [69]—or when the hormone is present in two different forms—e.g., total and non-active immunoglobulin-bound (macro) prolactins [70]. The method is performed in high-throughput format and helps to overcome shortcomings of separate detection. Another type of analysis requiring the detection of two targets is the single nucleotide polymorphisms (SNP) genotyping, when two allele variants have to be elicited simultaneously. Based on the colored obelin reporters, the microassay of products of the primer extension reaction during the SNP identification was also developed [69]. The method was successfully applied to identify: four SNPs associated with the risk of hemostasis disorders [71] and three SNPs in gene of melanocortin-1 receptor (MC1R), associated with melanoma and non-melanoma skin cancer risk [72,73]. All results were in complete agreement with those obtained with the use of conventional RT-PCR techniques or Sanger sequences, but bioluminescent analysis is faster and cheaper.

“Semi-synthetic” aequorin variants with altered emission profiles and decay kinetics have been developed through a combination of site-specific mutations and application of synthetic coelenterazine analogues [74]. In the study, two aequorin mutant proteins were genetically conjugated to three pro-inflammatory cytokines (tumor necrosis factor alpha (TNFα), interleukins IL6 and IL8). These fusion proteins were combined with *f-*, *i-*, and *cp-*coelenterazine, correspondingly (Figure 2). Fast bioluminescent signals from TNFα and IL8 were separated using bandpass filters with transmission peaks at 420 and 520 nm and measured within the first 6 s. The only signal left was emitted from the IL6 and it was integrated during the next 19 s. The validity of the assay was demonstrated by employing samples of artificial human serum, and the results were in agreement with those obtained by commercially available individual tests for each of the three cytokines.

The specificity of the binding assay is ensured by the combination of a reporter and biospecific molecule with affinity to a certain target (immunoglobulin, oligonucleotide, hapten, etc.). The molecules of the kind (biospecific bioluminescent labels) are usually obtained by chemical conjugation using different commercial reagents. To simplify and direct the synthesis, increase the conjugation yield, and to save the bioluminescence activity of photoproteins, several aequorin and obelin mutants carrying unique cysteine residues accessible for chemical modification were produced [75,76].

One of the modern trends in binding analysis is the use of DNA/RNA aptamers as the recognition and binding elements that can successfully compete with monoclonal antibodies. Aptamers are short single-stranded oligonucleotides with a unique spatial structure that enables them to bind target molecules with high affinity and specificity. They offer several unique advantages: a possibility of chemical synthesis with minimal batch-to-batch variation, long shelf life, stability, and a large variety of available chemical modifications. Moreover, aptamers can be selected for any target, including toxic and non-immunogenic ones. Due to these useful features, aptamers are becoming increasingly popular as biospecific elements in a number of biomedical analytical systems (see, for review, [77]).

Several bioluminescence aptamer-based analytical systems have been developed and tested on a large number of clinical samples. Obelin chemically conjugated with specific DNA aptamers was applied as a label in sandwich-type solid-phase analysis of lung tumor elements in clinical plasma. The developed assay demonstrated good sensitivity of 91.5% and specificity of 75% (*p* < 0.001) [78]. Analogous conjugate with (2′-F-Py) modified RNA aptamer to the multiple sclerosis (MS) related autoantibodies was used for detection of autoantibodies to myelin basic protein in sera samples from MS-diagnosed patients (91) and non-MS donors (81). A statistical analysis of the results showed a 63.7% sensitivity and a 94.2% specificity of the assay developed [79,80]. It is worth noting that the diagnosis and monitoring of MS requires sophisticated equipment and highly qualified personnel, and therefore, the development of an analytical approach is extremely relevant.

Using obelin as a reporter—a convenient technique—was developed for fast and easy monitoring of the DNA library enrichment and evaluating the affinity and specificity of the individual aptamers during the SELEX procedure [81]. The approach was applied for the selection and development of 2′-F-Py RNA aptamers to general and glycated hemoglobins—biomarkers of diabetes mellitus [82] and DNA aptamers to the cardiac troponin I—a specific biomarker for acute myocardial infarction [81].

In studies [83], the authors selected 2′-F-Py RNA aptamer to obelin and later used it for engineering structure-switching bi-modular aptamer constructs [84]. One aptamer module of the construct (antihemoglobin RNA aptamer) binds the analyte (hemoglobin) that causes structural reorganization of the second module making it able to bind the obelin molecule. The complex which is finally formed on the surface was detected by obelin bioluminescence. The proposed approach allows integrating the obelin reporter into the bioanalytical system without any chemical conjugation or engineering of the fused protein and might be further adjusted to the detection of other proteins simply by exchanging biospecific module.

Another way to connect the photoprotein molecule with biospecific polypeptide is to create a hybrid protein by fusing their gene. The approach has some benefits compared with chemical conjugation such as rapid bacterial production of homogeneous hybrids and easy purification using auxiliary affinity tags etc.

The fusion protein streptavidin to aequorin (STA-AQ) [85] and minimum-sized core streptavidin to obelin (SAV-OL) [86] were highly purified from the inclusion bodies in *Escherichia coli* (*E. coli*) cells and applied to a bioluminescent sandwich immunoassay. The obtained hybrids demonstrated several advantages over similar chemical conjugates: the easiest way of preparation; maximum retention of properties of the initial protein; and as a result, the higher sensitivity of the assay. The hybrid STA-AQ was tested in model immunoassay of α-fetoprotein, which is a serological marker of liver cancer. The SAV-OL was applied as a reporter for detecting several targets such as cardiac markers (troponin I, troponin T, myoglobin, creatine kinase MB) and nucleic acids. The hybrids containing immunoglobulin-binding polypeptide ZZ with aequorin or that with obelin were obtained recently [87,88]. The aequorin-containing construction included two auxiliary polypeptides: signal peptide sequence of the outer membrane protein A (OmpA) and hexahistidine tag, providing the hybrid translocation into periplasmic space of the host *E. coli* cells and fast purification by metal-affinity chromatography. The obelin-containing hybrid was much simpler without any auxiliary polypeptides and was purified from the inclusion bodies in a manner similar to wild-type obelin [89]. It has been shown to be a highly sensitive label in various assays: detection of antibodies, assessment of their affinity, interaction with recombinant proteins, monitoring of SELEX, and affinity assessment of DNA (RNA) aptamers [88].

The described types of fusion proteins are universal probes: hybrids STA-AQ and SAV-OL are probes to any biotin-containing molecule, hybrids ZZ-AQ and ZZ-OL are the probes to IgG due to protein A affinity to Fc fragments. Of note is that photoproteins can be extended with a polypeptide from N-terminus only, the extension of the C-end terminus induces photoprotein instability [90]. This is a significant limitation in designing the photoprotein hybrids.

Nevertheless, methods, materials, and approaches developed in the field allow the production of unique multifunctional proteins with new useful properties applicable in different areas. For example, a genetic engineering approach was used to construct bioluminescent molecular switch created by the insertion of specific proteins into the aequorin structure [91,92,93]. The hybrids developed in these studies included an aequorin molecule split into two parts (1–47 and 48–189 amino acid fragments) that were linked by either glucose-binding protein (GBP), cyclic AMP (cAMP) binding receptor protein (CRP), or a sulfate-binding protein (SBP). Target binding to the corresponding specific protein allosterically transduces AEQ, turning the switch “on” (in the case of GBP or SBP sensors) or “off” (in the case of cAMP) and generating or quenching bioluminescence emission. The developed molecular bioluminescent switches were validated by performing targets detection in clinical and environmental samples demonstrating selectivity and reproducibility with appropriate detection limits.

### 2.3. Analysis Based on the Aequorin Bioluminescence Inhibition

Another type of the analyte detection assay is based on the inhibition of aequorin bioluminescence activity upon its interaction with a small molecule.

In the study [94], aequorin bioluminescence inhibition assay for quantification of hydroxylated polychlorinated biphenyls (OH-PCBs), toxic and persistent environmental contaminants, was developed. The OH-PCBs, as the authors suppose, become bound to the hydrophobic CTZ-binding cavity of aequorin due to similarity with the structure of CTZ causing a dose-dependent decrease in aequorin bioluminescence. The proposed assay was applied for determination of various OH-PCBs in biological and environmental samples without their preliminary treatment. Rahmani H. et al. developed aequorin-based inhibition assay for direct detection of dopamine in raw samples of blood serum or urine with a detection limit of 53 nM [95]. The same research group proposed to use a aequorin bioluminescence system for superoxide anion detection [96]. The assay was based on decarboxylation of coelenterazine to coelenteramide by superoxide anion. The high superoxide anion concentration results in the increase in effective concentration of coelenterazine capable of charging the aequorin molecule. So, the bioluminescent signal of aequorin is inversely proportional to superoxide anion concentration with a linearity range between 4 and 40,000 pM and a detection limit of 1.2 pM.

One of the advantages of bioluminescence inhibition assay is the lack of aequorin modification step as it is both recognition and signal generating molecule. However, the researcher always must be sure that the sample does not contain other molecules, which, alongside with the target one, inhibit aequorin bioluminescence.

## 3. Photoprotein Pholasin and Its Analytical Application

Photoprotein pholasin is isolated from the luminous seawater mollusk *Pholas dactylus*. In 2000, apo-pholasin (34.6 kDa) was cloned and expressed in *E. coli* cells [97], although the main source of this photoprotein is the cultivation of *P. dactylus* in the farm of Knight Scientific Ltd. (Plymouth, UK) (see its application example, [98]). The luminescence reaction of pholasin is triggered by reactive oxygen species such as superoxide anion and hydroxyl free radical so it is now widely used as an indicator of the superoxide anion radical (SAR) and other reactive oxygen species (ROS) in various fields of research.

One of the recent pholasin applications is the quantification of leucocyte ROS production in response to various stimuli. Bryan and colleagues used the pholasin-based ROS assay to assess leukocyte respiratory burst response to biomaterials. They identified the role of a number of fabrication variables involved in the generation of tissue-based biomaterials in the down-stream leukocyte activation. A high degrees of leukocyte activation leads to poor material/patient compliance, accelerated degeneration, and graft rejection. The developed technique may be the way to guide host-material integration directed by the associated native tissue response to neutrophil ROS release and has the potential to be used as a routine component of presurgical evaluation to maximize foreign body compliance [99].

The same authors used pholasin to estimate the leukocyte ROS response for revealing differences in host reaction to several synthetic materials used in hernia repair [100]. Additionally, they utilized pholasin-based analysis to quantify leucocyte ROS production depending on the condition of the stem cell selection process [101].

Another pholasin application relates to studying the oxidative stress and inflammation during the disease course. Shah et al. used pholasin to evaluate superoxide anion (O_2_^-^) production in platelets of patients with heart failure [102]. Farthing et al. developed rapid and simple pholasin-based assay of inosine and hypoxanthine in human plasma, as potential biomarkers of acute cardiac ischemia [103]. The method has demonstrated the ability to distinguish between the healthy individuals, cases of non-traumatic chest pain, or potential acute cardiac ischemia. In study [104], the authors used pholasin for the measurement of whole blood antioxidant capacity at inflammatory processes, in particular in patients with rheumatoid arthritis and Parkinson’s disease. They found that the approach provides the information clarifying the role of oxidative stress in the development of the diseases.

## 4. CTZ-Dependent Luciferase Analytical Application

As noted above, CTZ-dependent luciferases catalyze oxidation of the substrate yielding its decarboxylated derivative (CTM) and a quantum of blue light. They are relatively small proteins with molecular masses ranging from 36 (luciferase from coelenterate *Renilla* (RLuc) [105]) to 16.5 kDa (one of the luciferase isoforms from copepod *Metridia longa* [106]). Luciferases, as opposed to Ca^2+^-regulated photoproteins, are not stable under chemical modifications and during the synthesis of biospecific conjugates can lose up to 60% of the initial bioluminescent activity (e.g., [107]). To overcome the problem, several original approaches were developed. So, the bioorthogonal conjugation or photoconjugation methods of obtaining luciferase-antibody conjugates that would provide a high conjugate yield, control over conjugation site and stoichiometry, and retention of the luciferase activity were elaborated [108,109]. However, the analytical application of luciferases in most cases is based on the bioluminescence of their derivatives extended with biospecific polypeptides obtained by genetic fusing. The *Renilla* luciferases, as the earliest studied ones, have been actively used as reporters, especially since 2006, when a variant RLuc8 with a significantly increased stability and bioluminescence yield was obtained using mutagenesis [110].

As a sensory component of hybrids, various biospecific polypeptides were applied—antibodies or their fragments [111,112,113,114], antigens [115,116,117,118], polypeptides with special binding ability [119,120,121,122], GFP and its color variants [123,124,125,126,127,128,129], etc. Based on the phenomenon of assembling split luciferase derivatives, a whole spectrum of homogeneous methods has been developed [125,126,127,128,129] and applied for studying protein–protein/ligand interaction [130,131,132,133], protein activity [134,135], and protein folding [136,137].

**Figure 5:**
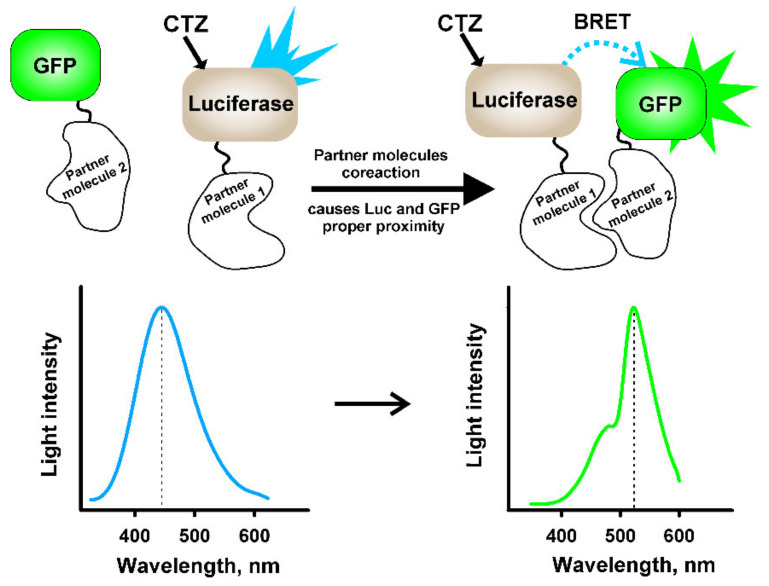
General principle of the BRET-based analytical systems.

The bioluminescence resonance energy transfer (BRET) assay (Figure 5) involving the use of RLuc has become popular since the late 1990s for measurements of protein–protein interactions and conformational rearrangements in live cells [138,139,140], for non-invasive bioimaging [141], and as probes for biosensing [123,142]. The sensitivity of BRET assays has recently been improved by introducing new BRET components: RLuc2 and RLuc8 with improved quantum yields, stability, and brightness [143] as well as a great variety of acceptors (GFP2, YFP, Venus, mOrange, TagRFP, TurboFP, semiconductor quantum dots or carbon-dots) [144,145,146]. However, the main application of RLuc is luciferase genetic reporter assay that has become an invaluable and routine tool for molecular biology research, including identification and characterization of protein functional variants [147,148], investigations of gene expression [148,149,150], transcription factors [151,152,153,154,155], receptor activity [156], miRNA expression [157,158,159], monitoring mRNA splicing events in cells [160,161], virus investigations [162,163,164], drug discovery [165,166], and identification and evaluation of virus inhibitors [167,168]. Moreover, RLuc is widely used for bioluminescence imaging of different processes in vivo [169,170,171] and guiding drug delivery to cells [172,173].

The luciferases of marine copepods *Metridia* and *Gaussia*, although their properties are still poorly studied, are attracting attention as promising reporters because of their small size, unique thermal stability, and genetically encoded secretion system. This makes them ideal reporters for in vivo applications by providing capability of monitoring cellular events via measuring bioluminescence in culture media without cell lysis (see recent comprehensive review, [174]).

Of special interest is the artificial luciferase developed recently and named anoLuc due to its small size of 19 kDa. It derived from the luciferase of deep-sea shrimp *Oplophorus gracilirostris.* Native luciferase consists of two heterodimeric polypeptides: a small subunit of 19 kDa (OLuc-19), which is responsible for bioluminescence activity but unstable in the absence of the 35 kDa subunit. Structural optimization of the OLuc-19 after 3-round random mutagenesis yielded a stable enzyme with 16 amino acids replacement. It produces glow-type luminescence with a specific activity almost 81,000-fold greater than that of the initial OLuc-19, and 5-fold of that of RLuc (under the same reaction condition) [17]. A new developed coelenterazine variant, reported in the same paper—furimazine (Figure 2)—enhances the bioluminescence level by almost 25 times compared to coelenterazine and provides long-term signal (t_1/2_ = 2.5 h) with a spectrum maximum at 460 nm. The commercial availability of NanoLuc, its cleaved variants capable of assembling with the restoration of bioluminescence, and furimazine have promoted a wide range of their applications as a versatile and flexible analytical tool. The review by England and coworkers of 2016 (only 4 years after the first publication on NanoLuc) highlighted five main areas of successful use of the system, namely, the study of protein–protein and protein–ligand interactions, the study of gene regulation and cell signaling, protein stability monitoring, and use as BRET-based biosensors and bioluminescence imaging [175]. The system has been becoming increasingly popular, and here, we focus on some recent publications describing fundamentally new research. In general, analytical systems based on NanoLuc reporters are used in the same research areas as RLuc ones, but they successfully compete with them due to the significantly lower molecular weight and commercial availability of various variants of this enzyme provided by Promega Co along with manuals. The prefix Nano is often added to the names of such systems (NanoBRET, NanoBiT, etc.).

### 4.1. NanoBit-Based Technologies

The NanoBiT approach is based on splitting NanoLuc into two separate subunits—large BiT (LgBiT, 18 kDa) and small BiT (SmBiT, 1.3 kDa). These subunits interact very weakly (K_D_ = 190 μM), so their assembly to form an active luminescent complex occurs only upon interaction of the separate binding partner proteins to which they are fused. In addition, the NanoBiT system includes subunit 11-amino-acid, HiBiT, with the essentially higher affinity to LgBiT (K_D_ = 700 pM). The protein of interest is genetically fused with HiBiT, and its expression is monitored by bioluminescence upon complementation with LgBiT in the presence of furimazine. So, while the SmBiT and LgBiT pair is applicable for detecting protein–protein interactions, the HiBiT acts as a detection tag for LgBiT.

**Figure 6:**
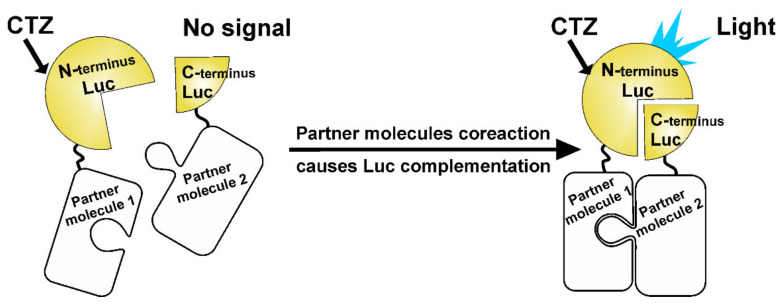
General principle of the assay based on the split luciferase complementation effect.

The system is successfully applied for protein–protein and protein–ligand (receptor) interaction research, extremely important in molecular biology (Figure 6). The most traditional version of the analysis is based on the use of radioactive tracers, which is technologically difficult and dangerous. In the paper of Soave et al., camelid single-domain antibody fragment (nanobody) VUN400, which recognizes the second extracellular loop of the human CXCR4 chemokine receptor, was used as a probe to monitor specific CXCR4 conformations. VUN400 was fused with HiBiT tag (VUN400-HiBiT) which complements LgBiT protein, forming a functional NanoLuc luciferase. Complemented luminescence was used to detect VUN400-HiBiT binding to CXCR4 receptors expressed in living HEK293 cells. [176]. A similar approach used the NanoBiT complementation for a high-throughput method to monitor the loss of adenosine A1 receptor (A1ARs) from the cell surface in living cells. In this study, the A1ARs was tagged on the N-terminus with HiBiT tag [177].

The NanoBiT system was applied to detect both strong and weak protein interactions such as those involving the binding of RAS oncoproteins to either RAF or phosphoinositide 3-kinase (PI3K) effectors, respectively, and was also shown to be effective for studying poorly soluble protein domains such as the RAS-binding domain of PI3K. [178].

Ubiquitination is a reversible post-translational modification of a protein that regulates many cellular processes (transcriptional signaling, protein degradation, protein transfer, and oncogenesis). To evaluate the ubiquitination processes, Boulch et al. fused SmBiT to ubiquitin and LgBiT to several ubiquitination substrates. The connection of ubiquitin to the substrate proteins of interest leads to reconstitution of NanoBiT reporter, and the corresponding luminescence signals are recorded with a microtiter plate reader. This approach is applicable to study ubiquitination in tissue culture systems or genetically amenable model organisms [179].

The Hippo signaling pathway regulates a variety of biological functions, and its dysregulation triggers the development of different cancer types. The Hippo pathway negatively regulates the activity of the transcriptional coactivators YAP and TAZ. TEAD is the main transcription factor that mediates YAP/TAZ oncogenic functions. It has been proposed that the disruption of the YAP/TAZ–TEAD interaction would be a way to restrain the transcriptional outputs of YAP/TAZ for cancer treatment. Using a NanoBiT system, a number of fuses containing NanoLuc fragments and YAP/TAZ or TEAD in different consequences were obtained and expressed in HEK293T cells. The combination of SmBiT–YAP/TAZ and LgBiT–TEAD demonstrated the highest sensitivity and dynamic range of analysis. The obtained YAP–TEAD and TAZ–TEAD biosensors were then successfully used in high-throughput small molecule drug screening [180].

The application of NanoBiT technology in binding assay in vitro is described in Section 4.3.

### 4.2. NanoBRET-Based Technologies

NanoLuc as the energy donor in BRET assay, termed nanoBRET [181], offers great application potential due to its obvious advantages over other BRET luciferases. First of all, the high quantum yield of NanoLuc and the possibility to combine BRET assay with NanoLuc-derived split luciferase reporter system (NanoBiT, described above) ensures the development of BRET assay with increased sensitivity. It allows the monitoring of protein–protein interactions at lower and more physiologically-relevant levels [182] and analysis of interactions with low selectivity or affinity which have previously been challenging [183,184]. The NanoLuc bioluminescence spectrum (λ_max_ = ~460 nm) is about 20% narrower compared with that of RLuc, enabling improved spectral separation of donor and acceptor emissions and the use of various fluorophores as acceptors. The numerous studies have demonstrated nanoBRET to utilize a broad range of fluorophores—from different fluorescent proteins (CyOFP, red-shifted CyOFP1, mNeonGreen, mScarlet, mCitrine, TurboFP635, mOrange, TagRFP, etc.) [185,186,187,188,189,190] to fluorescent dyes (TAMRA, BODIPY, 4-nitro-7-aminobenzofurazan (NBD), Alexa Fluor^TM^, etc.) [191,192,193,194,195]. This greatly expands characteristics of the nanoBRET spectrum and also makes the development of microscopic nanoBRET imaging possible [195]. Fluorescent dyes in this case are conjugated to HaloTag protein (HaloTag system, see [196]). Small size of NanoLuc and its capacity to fold appropriately in extracellular environments has provided reason for its use in receptor–ligand binding studies where NanoLuc is fused to the extracellular N-terminus of the receptor without impairing localization at the plasma membrane [197]. Moreover, when used as a fusion reporter, NanoLuc is theoretically less likely to cause function-altering steric hindrance. This allows it to be used in BRET assays where the possibility of changing the function of a fusion structure can be a concern [198]. Comprehensive reviews on NanoBRET methodology and applications have been published recently [197,198]. NanoBRET is successfully used for studying protein–protein interactions [184,199], or investigations on receptor activation [190,199,200], oligomerization [201], conformational states [186], proximities [189,202], and receptor–ligand interactions [200,201,203]. NanoBRET technology has been also applied for in vivo imaging. For example, Min and colleagues generated a ratiometric BRET sensor of ATP for in vivo imaging of metabolic status [188]. Ong et al. developed a pH-sensitive nanoBRET sensor and demonstrated its diagnostic value for pH variations across the tumor microenvironment [204]. Taylor et al. showed NanoBRET in combination with firefly luciferase to unambiguously discriminate two cell populations in vivo with high sensitivity in the same imaging session [205]. To obtain red spectral emission, optimal for in vivo imaging with applicable BRET efficiency, appropriate acceptors were selected [195,206] and various synthetic coelenterazine analogues were utilized [207,208,209]. Additionally, based on nanoBRET technology, some genetically encoded calcium ion indicators were developed [210,211] and used for imaging Ca^2+^ concentration changes in cultured cells and neurons [211].

Due to the remarkable increase in bioluminescence activity offered by NanoLuc, combined with its high thermal stability and pH-insensitivity, NanoLuc-based BRET sensors have started to emerge as an attractive sensor format for use in point-of-care applications. Recently, nanoBRET sensor platforms for detection of small-molecule drugs (LUCiferase-based Indicators of Drugs, LUCIDs) and antibodies (LUMinescent AntiBody Sensor, LUMABS) have been developed by the groups led by M. Merkx. Sensing mechanisms of LUCIDs and LUMABS sensors are described in detail in [212,213]. These sensors allow measurement of antibodies directly in blood plasma, eliminating the need for the complex liquid handling steps associated with conventional immunoassays [214]. Bioluminescence can be easily registered with a smartphone digital camera [215]. The developed sensors were applied to detect anti-HIV1, anti-hemagglutinin, and anti-dengue virus type I antibodies [212,214,215] as well as methotrexate, dinitrophenol, creatinine, and theophylline molecules [212,213,216]. In principal, the sensor’s modular design allows the easy adaptation to many other analytes. Engelen W. et al. developed a NanoBRET-based protein-DNA hybrid molecular beacons sensor that permits the detection of low pM concentrations of nucleic acids (exemplified by miRNA21) directly in complex media. Due to the use of bright NanoLuc, these BRET beacons are at least 2 orders of magnitude more sensitive than the previously reported nucleic acid BRET sensors [217]. Moreover, nanoBRET-based homogeneous insulin assay was developed by Shigeto and colleagues [218]. The BRET signal was proportional to the insulin concentration, and the lower detection limit was 0.8 μM. An important step towards the application of LUCID, LUMABS, and other BRET-based diagnostic assays is the translation from a laboratory-based assay to a fully implemented point-of-care assay. The assay reported by Shigeto can be also applied to continuous monitoring of the time-dependent cellular response in vitro and as authors expected to drug development by high-throughput screening [218].

### 4.3. Application of NanoLuc as a Label for In Vitro Binding Assay

Similar to the other luciferases, NanoLuc loses its bioluminescent activity upon chemical conjugation with biospecific molecules: we could not find any published work regarding the preparation of such conjugates. Therefore, NanoLuc tags for in vitro binding assay are typically produced by genetic fusion with biospecific polypeptides—antibodies or antigenes. For example, Ren et al. genetically fused NanoLuc with aflatoxin-specific nanobody, obtained from an immune alpaca phage-display VHH library. The fusion protein was applied as a label in one-step competitive bioluminescent immunoassay for the detection of aflatoxin in cereal, which showed more than a 20-fold improvement in sensitivity as compared to classical ELISA [219]. NanoLuc fused with HIV-1 p24 or gp41 antigen was applied for qualitative and quantitative detection of anti-HIV-1 antibody in sera with the sensitivity exceeding that of ELISA 104-fold [220]. A similar assay was used to detect human norovirus-specific antibodies [221] or autoantibodies directed against proinsulin/insulin, which is important for diagnosis of type 1 diabetes [222].

Genetic fusion has the advantage of generating homogeneous conjugates with a well-defined antibody-luciferase stoichiometry. However, it requires cloning for each new antibody-luciferase conjugate and often involves cumbersome expression optimization and access to mammalian expression systems. Wouters et al. reported an easily accessible, efficient, and chemoselective method for the synthesis of antibody-luciferase conjugates. [109]. The NanoLuc was genetically fused with protein G domain containing the photo-cross-linkable non-natural amino acid para-benzoylphenylalanine (developed by Hui et al. [223]) that can be photo-cross-linked to the antibody using UV light illumination (365 nm). The method does not require cloning or recombinant antibody expression and can be directly applied to almost all human and many mammalian monoclonal IgG antibodies. The obtained NanoLuc-antibody conjugates were used in cell immunostaining, solid-phase immunoassay, and Western blotting [109].

The excellent thermostability of NanoLuc allows the obtaining of protein-based nanoparticles displaying multivalent NanoLuc on their surfaces. NanoLuc was genetically fused with elastin-like polypeptides containing poly-aspartic acid tail (ELP-D). Above the transition temperature (40 °C), the ELP-D forms aggregates whose sizes are regulated to around 30 nm as a result of the charge repulsion of the poly-aspartic acid chains. These particles displaying multiple molecules of NanoLuc were used as a detection probe in immunoassay of α-fetoprotein. The sensitivity of the developed assay was 10 times higher than that of an assay relying on the use of the monomeric version of the fusion protein [224].

The original approach to obtain NanoLuc conjugate with DNA fragments was described in [225]. Luciferase was genetically fused with the catalytic domain of replication initiator protein (pRep) from porcine circovirus type 2. The trombin DNA aptamer elongated with the pRep recognition oligonucleotide was synthesized chemically. Stable non-covalent complex DNA-NanoLuc was obtained by mixing these two compounds and examining as a label in model trombin sandwich solid-phase assay, showing the excellent detection limit of 0.1 nM. The value of this method is that, in principle, it can be applied for the synthesis of many other DNA-luciferase complexes.

The T7 coliphage was genetically engineered to express the NanoLuc, and was used as a bioluminescent indicator of bacterial contamination [226]. In this construct, NanoLuc was fused to the carbohydrate binding module (CBM), providing specific binding of the reporter to crystalline cellulose. The engineered phage infected *E. coli* in the water sample (if any), resulting in NanoLuc-CBM expression. It was irreversibly immobilized on cellulose particles, which were added into the sample, and collected by centrifugation or fixed on a cellulose membrane by filtration. The reporter bioluminescence was measured on the addition of a substrate. The phage-based assay demonstrates its significant diagnostic value for inexpensive, rapid, and sensitive detection of *E. coli* in drinking water [226]. Due to the inherent specificity of phages for the respective bacteria, an approach can be developed to detect other bacterial contaminants. A similar approach was used to determine bacterial contamination in cheese [227].

Of special interest is the application of NanoBiT technology for in vitro binding assay. Even though bioluminescent reporters based on the luciferases of firefly, click beetle, or *Renilla* were split and extensively used as excellent reporters that display sensitive and reversible signals, these luciferases are not very stable and their reconstitution efficiency is not very high—typically less than 1%—as assumed by an in vitro experiment using purified probes (e.g., [228]). For this reason, the application of luciferase protein fragment complementation assay to in vitro diagnostics has been hampered in spite of its potentially wide applications as a sensitive homogeneous assay. Ohmuro-Matsuyama and Ueda have developed a homogeneous non-competitive open sandwich bioluminescent immunoassay of osteocalcin peptide using NanoLuc-based protein fragment complementation assays [229]. They additionally split LgBiT into two parts, yielding a smaller N-terminal derivative (LnBiT) and two C-terminal, 11-residue peptides (LcBiT and SmBiT) corresponding to consecutive beta strands. These fragments were fused with variable regions V_H_ and V_L_ of anti-osteocalcin antibody, correspondingly. When LnBiT, V_H_-LcBiT, and V_L_-SmBiT were mixed, the bioluminescence intensity increased remarkably depending on the dose of osteocalcin peptide. The EC50 value and the detection limit were 123 ± 16 nM and 5 nM, respectively. Since the bioluminescent signal was strong and stable enough and could be observed with the naked eye or digital camera, it could become the foundation for developing many other point-of-care detection systems.

Ranawakage et al. used a NanoLuc-based HiBiT system to develop quantitative immunoprecipitation assay for determining the dissociation constant (Kd) of antigen–antibody interactions in solution [230]. The assay applicability was demonstrated by examining the affinities of monoclonal antibodies against the epitope tags (FLAG, HA, V5, PA, and Ty1).

Tetsuo and colleagues developed a novel high-throughput serum neutralization test (SNT) for the serological diagnosis of infections (classical swine fever, bovine diarrhea, and borderline disease viruses) based on genetically engineered pestiviruses expressing the HiBiT peptide [231]. Swine or bovine kidney cells line were infected with recombinant viruses in serially diluted antiserum. Recombinant virus growth in cell culture occurs if the serum does not contain neutralizing antibodies. The luciferase activity of culture supernatants or cell lysates was measured using a Nano-Glo HiBiT lytic detection system (Promega, Madison, WI, USA). It depended on serum dilution and the time of virus inoculation. The authors concluded that the luciferase-based SNT will replace conventional SNTs. The proposed method is suitable for high-throughput testing for the control and study of pestiviruses.

### 4.4. NanoLuc in Genetic Reporter Assay

NanoLuc application in luciferase genetic reporter assay does not fundamentally differ from the RLuc one. What is essentially new here is that due to the small size and extreme brightness of NanoLuc, it is widely used as an effective reporter in developing stable reporter viruses. These luciferase-tagged viruses are applicable not only for studying the virus life cycle in vitro and in vivo (viral proliferation, replication, assembly/release and entry) and analyzing virus-host interactions but also for evaluating therapeutic strategies and developing new inhibitors and vaccines. NanoLuc-based reporter viruses were successfully designed to study such dangerous diseases as: influenza A virus [232,233,234,235,236], hepatitis B [236,237,238,239] and E viruses [240], HIV [241], rotavirus [242], flavivirus [243,244,245], and arboviruses [246] which indicates the great potential of NanoLuc for such application.

In the recent years, CRISPR/Cas9 has emerged as a prominent technology for genome editing due to simplicity and ease of use. There are some publications describing the integration of NanoLuc gene directly into the endogenous gene using the CRISPR/Cas9 system. This allows efficient measurement of transcriptional activity for genes of interest in native epigenetic landscape which is not achievable under exogenous transfection-based luciferase reporter assays. The novel approach may provide new insights into understanding precise gene regulation in various developmental and pathophysiological conditions [247,248]. Moreover, the NanoLuc-based CRISPR/Cas9 system is a very useful tool to isolate gene-edited cells [248,249,250]. By combining the HiBiT reporter technology with the CRISPR/Cas9 editing, Schwinn et al. [251] developed the approach for sensitive quantification of both protein abundance and its post-translational modifications.

## 5. Conclusions

**Figure 7:**
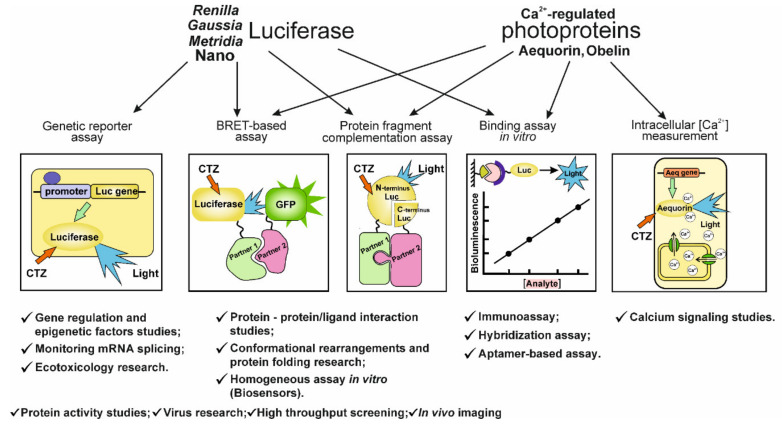
Main principles on the basis of which analytical methods and investigations can be carried out.

The analysis of the literature data on the use of coelenterazine-dependent luciferases as bioluminescent reporters clearly shows them to be a powerful analytical tool capable of solving a wide range of research and application problems. Figure 7 lists the main principles of analytical methods and the investigations that can be carried out on their basis. Some approaches are implemented involving both luciferases and Ca^2+^-regulated photoproteins, whereas some of them can be applied considering the unique capabilities of the reporters belonging only to each of these two groups. A number of approaches have become common and widely used, while others hold much promise for development analytics urgently needed for the present moment. A case in point here is the NanoLuc-based analytical system that Promega (USA) challenged to propose for the rapid detection of antibodies to SARS-Cov2 virus [252]. 

A huge number of various enzymes and their substrates, including those artificially created and commercially available, which have various useful properties, provide a real opportunity for the experimenter to choose the option that best suits his needs. Within the framework of this review, we did not mention the availability of modern photometers—their variety and high analytical characteristics are able to satisfy the highest requirements of the users. Analytical approaches and methods based on these bioluminescent systems make it possible to successfully solve not only purely fundamental problems of modern biology. Their widespread introduction into practice of biomedical and environmental laboratories is also evident.

## Abbreviations

CTZ	coelenterazine
CTM	coelenteramide, decarboxylated derivative of CTZ
Luc	luciferase
PhP	Ca^2+^-regulated photoprotein
apoPhP	polypeptide part of PhP
CBP	Ca^2+^-dependent coelenterazine-binding protein
apoCBP	polypeptide part of CBP
GFP	green fluorescent protein
EF-hand	The Ca^2+^-binding sites formed by helix-loop-helix motifs
